# Expanding the therapeutic scope of PARP inhibitors in breast cancer: current indications and future directions

**DOI:** 10.1172/JCI207632

**Published:** 2026-10-01

**Authors:** Charlotte S. Walmsley, Adela Rodriguez, Panagiotis A. Konstantinopoulos, Geoffrey I. Shapiro, Sara M. Tolaney, Judy E. Garber, Filipa Lynce

**Affiliations:** 1 Department of Medical Oncology, Dana-Farber Cancer Institute, Boston, Massachusetts, USA.; 2 Harvard Medical School, Boston, Massachusetts, USA.; 3 Division of Cancer Genetics and Prevention, Dana-Farber Cancer Institute, Boston, Massachusetts, USA.

## Abstract

Homologous recombination repair deficiency (HRD) occurs in approximately 10% of breast tumors and represents a major targetable vulnerability across multiple cancer types. Impairment of the homologous recombination DNA repair pathway — arising through somatic and germline mutations in homologous recombination repair genes, epigenetic mechanisms, and transcriptomic changes — leads to genomic instability and a reliance on error-prone repair mechanisms. Poly (ADP-ribose) polymerase (PARP) inhibitors harness the synthetic lethality of HRD and PARP inhibition, with current approvals encompassing both early-stage and advanced breast cancer in patients with germline *BRCA1/2* (g*BRCA1/2*) mutations. However, emerging evidence suggests efficacy of PARP inhibitors beyond g*BRCA1/2*-mutated breast cancer to tumors with other homologous recombination repair defects that confer a “BRCAness” phenotype. This Review examines opportunities to broaden the use of PARP inhibitors in breast cancer and underscores the importance of innovative biomarkers, combination strategies, and next-generation agents to maximize therapeutic impact.

## Introduction

Homologous recombination is critical for maintaining genomic integrity and supporting efficient DNA repair (1). Pathogenic variants in multiple genes within the homologous recombination pathway, including *BRCA1*, *BRCA2*, and partner and localizer of *BRCA2* (*PALB2*), have been implicated in increased inherited and acquired breast cancer risk (2). Among these, germline mutations in *BRCA1* and *BRCA2* remain the most common and are associated with aggressive tumor biology and younger age of onset (3).

Poly (ADP-ribose) polymerase inhibitors (PARPis) are approved by the US FDA for patients with early-stage and advanced breast cancer and germline *BRCA1* and *BRCA2* (g*BRCA1/2*) deleterious mutations. A growing body of evidence demonstrates that PARPis exert antitumor activity beyond g*BRCA1/2* mutations, including in cancers harboring other homologous recombination repair (HRR) gene alterations, somatic *BRCA1/2* (s*BRCA1/2*) mutations, genome-wide loss of heterozygosity (LOH), and elevated genomic instability scores (4–6). Expanding PARPi use beyond g*BRCA1/2*-mutated disease remains an active focus of investigation.

This Review examines the evolving role of PARP inhibition in breast cancer, spanning established use in g*BRCA1/2*-mutated disease, emerging evidence beyond g*BRCA1/2* alterations, the development of next-generation PARPis and combination strategies, and key future directions shaping the field.

## PARPi use in g*BRCA1/2*-mutated breast cancer

*BRCA1* and *BRCA2* have multiple critical roles in maintaining genomic integrity, including repairing double-strand DNA breaks through HRR, protecting replication forks, and preventing replication fork–associated gaps (Figure 1) (7–11). Germline mutations in *BRCA1/2* result in hereditary cancer syndromes with elevated risks of breast cancer, ovarian cancer, and multiple other malignancies (12, 13). Defective DNA repair leads to genomic instability, cell cycle dysregulation, and unchecked proliferation. However, it also creates a therapeutic vulnerability that can be exploited by targeted anticancer therapies.

Poly-ADP-ribosylation is a critical process for multiple DNA repair functions, including the recruitment and regulation of key base excision repair factors, such as DNA polymerase-β, XRCC1, and DNA ligase IIIα, as well as the promotion of chromatin relaxation (14–16). Poly-ADP-ribosylation is carried out by enzymes belonging to the ADP-ribosyltransferase superfamily, including PARP1 and PARP2. The primary targets of all currently approved PARPis, PARP1 and PARP2, are activated upon DNA damage to function as posttranslational modifiers within the BER pathway (17–19).

PARPis impair DNA damage repair pathways by targeting PARP enzymes, ultimately leading to cell death in HRR-deficient (HRD) tumors (20, 21). Multiple mechanisms have been proposed to underlie the synthetic lethality between PARP inhibition and *BRCA1/2* deficiency, including inhibition of PARP-mediated BER (22), trapping of PARP-DNA complexes leading to replication fork collapse (23), and accumulation of single-strand DNA gaps at replication forks (24). Two PARPis, olaparib and talazoparib, are FDA approved for the treatment of breast cancer.

For early-stage disease, FDA approval of olaparib was based on results from the randomized phase III OlympiA trial, which evaluated 1 year of adjuvant olaparib versus placebo in patients with g*BRCA1/2* mutations and high-risk human epidermal growth factor receptor 2–negative (HER2-negative) breast cancer. At 4 years of follow-up, olaparib significantly improved invasive disease-free survival (82.7% vs. 75.4%; HR 0.63), distant disease-free survival (86.5% vs. 79.1%; HR 0.57), and overall survival (OS) (89.8% vs. 86.4%; HR 0.68; *P* = 0.01), compared with placebo (25).

In the OlympiAD trial, olaparib significantly prolonged progression-free survival (PFS) compared with chemotherapy (7.0 vs. 4.2 months; HR 0.58; *P* < 0.001) in patients with metastatic breast cancer (MBC) harboring g*BRCA1/2* mutations (26), without a corresponding OS benefit (27). However, a follow-up analysis suggested a greater OS benefit when using olaparib in the first-line setting compared with chemotherapy (22.6 vs. 14.7 months; HR 0.51; 95% CI: 0.29–0.90) (28). Similarly, in the EMBRACA trial, talazoparib demonstrated a 3-month PFS benefit compared with standard chemotherapy (8.6 vs. 5.6 months, HR 0.54, *P* < 0.001), with significant improvements in patient-reported quality of life and breast symptoms, and a numeric but not significant OS benefit (29). The pattern of PFS benefit without a corresponding OS benefit in the metastatic setting may be explained by underpowered OS analysis, subsequent postprogression therapies, and, particularly in EMBRACA, subsequent PARPi use in the chemotherapy arm (27, 29). Both agents were generally well tolerated, with adverse events rarely leading to treatment discontinuation. Olaparib was associated mostly with gastrointestinal symptoms, fatigue, and hematologic toxicities. Talazoparib was associated with a higher incidence of severe hematologic adverse events, likely due to higher PARP-trapping activity (27, 30).

## PARPi use beyond g*BRCA1/2-*mutated breast cancer

HRD can arise through several mechanisms, including inherited germline variants, acquired somatic mutations, epigenetic alterations, and transcriptomic changes of HRR pathway genes (31). Efforts to better define and identify “BRCAness,” a state of HRD in tumors without g*BRCA1/2* mutations, has emerged as a major area of investigation with important therapeutic implications (Figure 2) (32).

s*BRCA1/2* mutations occur spontaneously in approximately 3% of breast cancers (33–35). These somatic mutations closely resemble g*BRCA1/2* mutations in their mutation type (including deletions, insertions, single nucleotide variants, and exon deletions), as well as degree of biallelic inactivation, genome-wide LOH, large chromosomal events, and telomeric imbalance (34–38). However, s*BRCA1/2* mutations occur less frequently than g*BRCA1/2* mutations and are generally diagnosed in older patients (35).

Growing evidence suggests efficacy of PARPis in patients with s*BRCA1/2* mutations and other HRR deficiencies (Supplemental Table 1; supplemental material available online with this article; https://doi.org/10.1172/JCI207632DS1). The phase II Olaparib Expanded trial evaluated olaparib in MBC patients with s*BRCA1/2* mutations or germline mutations in other HRR genes. For patients with s*BRCA1/2* mutations, the study reported an objective response rate (ORR) of 50% with a median PFS (mPFS) of 6.3 months. Analysis of an expansion cohort confirmed sustained activity, with an ORR of 37% (11/30) and a mPFS of 5.5 months (39, 40). Similar findings were shown in a small cohort of patients with metastatic triple-negative breast cancer (TNBC) (41).

Additional prospective data are anticipated from an ongoing phase II trial evaluating talazoparib in MBC patients with s*BRCA1/2* mutations (42). In line with new findings, the National Comprehensive Cancer Network Clinical Practice Guidelines for Breast Cancer were updated to include a statement on the potential efficacy of olaparib for s*BRCA1/2* mutations in patients with MBC (43).

### Mutations in other HRR genes.

With the rapid expansion of germline and somatic tumor genetic testing, attention has shifted beyond *BRCA1/2* to other HRR gene alterations as potential biomarkers of PARPi sensitivity. Among these is *PALB2*, which assists in the nuclear localization and stability of *BRCA2*, thus enabling its homologous recombination and checkpoint functions (44, 45). Inactivation of *PALB2* confers HRD, leaving tumors vulnerable to PARP inhibition (46).

Consistent with this biological rationale, early clinical studies have shown meaningful activity of PARPis in patients with germline *PALB2* (g*PALB2*) mutations, though available data remain limited. In the phase II Olaparib Expanded trial, 11 patients with g*PALB2* mutations achieved a mPFS of 13.3 months and an ORR of 82%, with similarly favorable outcomes in the expansion cohort (PFS 9.6 months, ORR 75%) (39). Additional evidence comes from the Talazoparib Beyond BRCA trial, which evaluated talazoparib in patients with solid tumors and wild-type *BRCA1/2* harboring mutations in other HRR genes. Among 6 patients with g*PALB2* mutations, the mPFS was 6.9 months with an ORR of 50%, and all 5 patients with breast cancer achieved tumor shrinkage as best response (47).

In contrast, clinical activity of PARPis in tumors harboring mutations in other HRR genes appears less consistent (48). In the Olaparib Expanded Trial, no responses were observed in patients harboring *CHEK2* (*n* = 10) or *ATM* (*n* = 11) mutations (40). A study from Hinic and colleagues showed that CHEK2-deficient tumors do not exhibit mutational signatures consistent with HRD, offering a mechanistic explanation for the absence of meaningful PARPi activity in this population (48). The lack of benefit observed in patients with *ATM* mutations may be explained by the variability of these mutations, particularly monoallelic or missense mutations, to confer ATM protein loss, as well as the compensatory pathways capable of maintaining homologous recombination (49, 50). However, preclinical evidence in other disease groups suggests ATM-deficient tumors may be more sensitive to PARPis in the appropriate circumstances, including when combined with ATR inhibition (51), or in the presence of an *ASXL1* mutation (52), highlighting the complexity of translating HRR genotypes into clinically actionable biomarkers. Despite this, clinical studies have not demonstrated a clear benefit in breast cancer (53).

### HRD genomic scar assays.

Beyond somatic and germline mutations in HRR genes, multiple additional mechanisms can cause HRD. This complexity has driven efforts to create technologies that capture HRD across the genome aimed at predicting sensitivity to PARPis. Tumors with HRD depend on error-prone alternative pathways to resolve double-strand DNA breaks, resulting in characteristic genomic features, including large-scale chromosomal rearrangements detectable by genomic scar assays and distinct somatic mutational patterns identified through mutational signature profiling (54, 55).

Genomic scarring associated with HRD is measured through three primary mechanisms: LOH, TAI, and LSTs (56–58). Abnormalities in each of these categories can be quantified and integrated to generate a composite assessment of HRD. Multiple clinical assays have been developed to detect these genomic signatures of defective DNA repair, including the myChoice CDx test by Myriad Genetics and the FoundationFocus CDx_BRCA LOH_ test (59, 60).

The myChoice HRD test includes *BRCA1* and *BRCA2* gene sequencing and quantification of LOH, TAI, and LST to create a Genomic Instability Score. This assay is FDA approved in ovarian cancer to guide the use of olaparib with bevacizumab in the first-line maintenance setting (61–66). In breast cancer, Telli and colleagues demonstrated that a combined HRD score — including LOH, TAI, and LST — significantly predicted pathologic complete response (pCR) to neoadjuvant platinum chemotherapy in patients with early-stage TNBC, even those without *BRCA1/2* mutations (61). The FoundationFocus CDx_BRCA LOH_ test, which integrates LOH scoring with HRR gene mutation analysis, was used in the ARIEL2 and ARIEL3 trials in patients with ovarian cancer to study the efficacy of the PARPi rucaparib (67, 68). In the ARIEL2 trial, patients with *BRCA1/2* mutations or high LOH scores achieved a significantly longer PFS with rucaparib compared with those with low LOH scores. Thus far, neither assay has received FDA approval as a companion diagnostic to guide PARPi use in breast cancer, though this remains an area of strong clinical interest.

### HRD mutational signatures.

Somatic mutation analysis and HRD mutational signatures represent an alternative approach to quantifying HRD and identifying patients who may be sensitive to PARPis. HRDetect is a mutational signature–based test that combines six genomic signatures derived from high-depth whole-genome sequencing data to predict tumor BRCAness (69). Development of the HRDetect model consisted of initial training with g*BRCA1/2*-null samples used as positive controls, followed by validation with independent cohorts of breast, ovarian, and pancreatic tumor samples. In a cohort of 560 breast cancer tumors, the HRDetect test identified all 22 patients with g*BRCA1/2* mutations, as well as 22 patients with s*BRCA1/2* mutations and 47 tumors with functional BRCA1/2 deficiency without identifiable mutations. The phase II RIO trial evaluated rucaparib in 43 patients with untreated TNBC and demonstrated greater PARPi sensitivity in patients classified as HRD positive by HRDetect testing, as reflected by decreased circulating tumor DNA levels following treatment (70).

Additional clinical evidence supporting HRD-associated mutational signatures as predictive biomarkers of PARPi sensitivity comes from a retrospective cohort study by Batalini and colleagues. Using a clinico-genomic database linked to clinical outcomes, the investigators analyzed 177 patients with MBC treated with PARPis and found that patients with HRD-positive mutational signatures experienced significantly longer PFS than their HRD-negative counterparts (6.3 vs. 2.8 months; HR 0.62, 95% CI 0.42–0.92) (71). While these findings support its potential utility, the HRDetect algorithm has yet to be prospectively validated as a biomarker for predicting PARPi response, and its reliance on whole-genome sequencing may limit widespread implementation in routine clinical practice (72).

A major limitation of mutational signatures and genomic scar assays is their insensitivity to known mechanisms of PARPi resistance. For instance, when HRR proficiency is restored through secondary reversion mutations or DNA end resection rewiring, the genomic alterations accumulated during the period of HRD remain embedded in the tumor genome (73, 74). As a result, scar-based and mutational signature assays may continue to classify these tumors as HRD despite functional restoration of HRR proficiency (75). Functional biomarkers that directly assess real-time HRR competency, including transcriptome signatures and RAD51 foci formation assays (74), may help overcome this limitation by more accurately capturing dynamic changes in DNA repair capacity.

### Transcriptome signature/gene expression profiling.

By measuring functional HRR status, transcriptomic assays may better capture the dynamic nature of BRCAness, including adaptive changes associated with treatment resistance and tumor progression. Multiple signatures have been developed to quantify transcriptional HRD (76–78). In an early proof-of-concept study, Konstantinopoulos and colleagues used a microarray gene expression profile to characterize BRCA-like and non-BRCA-like tumors in 61 patients with epithelial ovarian cancer and demonstrated an association between BRCA-like expression and sensitivity to platinum chemotherapy and PARPis (79). Notably, 2 of 4 patients who underwent longitudinal sample collection exhibited alterations in their BRCAness profiles concomitant with the development of platinum resistance during treatment, underscoring the relevance of real-time evaluation of HRD status.

In breast cancer, Kang and colleagues developed a transcriptomic HRD signature that outperformed genomic HRD assays and *BRCA1/2* mutation status in predicting sensitivity to multiple PARPis across cell lines and patient cohorts (78). These findings were further supported by results from an arm of I-SPY2, a phase II adaptive randomized clinical trial that used a gene expression–based diagnostic signature to identify tumors with a BRCA1ness phenotype. In the neoadjuvant setting, patients received either an oral PARPi combined with chemotherapy or standard chemotherapy alone. The study demonstrated a significant association between BRCA1ness and pCR, with the BRCA1ness signature predicting a higher likelihood of pCR in the veliparib–carboplatin arm (*P* = 0.03; OR, 3.2) but not in the carboplatin-alone arm (*P* = 0.45) (80). Although these studies demonstrate encouraging associations between transcriptomic HRD signatures and therapeutic response, additional validation in prospective clinical trials is necessary to establish their role in guiding PARPi therapy.

### Protein expression.

Protein levels of DNA damage response genes, particularly ATM, have been studied using immunohistochemistry (IHC) as a surrogate marker for HRD status (81). In metastatic gastric cancer, a clinical trial assessing combination therapy with a PARPi and paclitaxel demonstrated improved OS among patients whose tumors lacked ATM protein expression by IHC compared with those retaining ATM expression (82). However, follow-up analysis failed to reproduce this survival benefit (83). To date, similar IHC-based studies have not been systematically conducted in breast cancer populations, in part because IHC analysis has increasingly been replaced by other approaches for HRD characterization.

### Functional assays: RAD51 foci detection.

The RAD51 foci assay has emerged as the most clinically utilized and validated functional test for predicting PARPi response in breast cancer. RAD51 is a critical recombinase protein for HRR, forming RAD51 nucleoprotein filaments at sites of DNA double-strand breaks during the S or G2 phase of the cell cycle. The absence of RAD51 foci formation after DNA damage is indicative of deficient HRR (84).

Several testing strategies have been developed to assess RAD51 foci in breast cancer, including fresh tissue approaches, FFPE tissue–compatible tests, liquid biopsies, patient-derived xenograft–based (PDX-based) testing, and molecular profiling (85–89). Pellegrino and colleagues evaluated HRR genes, a genomic HRD test, and RAD51 foci detection across 103 breast cancer PDX models. RAD51 foci assessment was the most accurate predictor of PARPi response, achieving a predictive accuracy of 95% compared with 71% for genomic HRD analysis and 67% for HRR gene mutation analysis (89). Consistent findings were reported by Castroviejo-Bermejo and colleagues, in which RAD51 scores tracked more closely with PARPi response than genomic HRD scores in breast cancer PDX models (90).

Supporting the clinical relevance of RAD51, a post hoc biomarker analysis of the phase II GeparOLA trial in patients with early-stage HER2-negative breast cancer receiving neoadjuvant PARPi therapy combined with paclitaxel demonstrated that low RAD51 score was associated with higher rates of pCR to PARPi-based or platinum-based therapies (OR 12.03, 95% CI 2.60–55.73, *P* = 0.002) (91). Similarly, the phase II RADIOLA trial evaluating olaparib in patients with HER2-negative MBC showed a substantial improvement in ORR in RAD51-low compared with RAD51-high tumors (68.3% vs. 21.4%, *P* = 0.002) among patients harboring germline or somatic *BRCA1/2*, *PALB2*, or *RAD51C/D* mutations (92).

As a functional test that theoretically detects real-time HRR status, RAD51 foci detection is also an intriguing strategy for predicting PARPi resistance in patients who may have restored HRR functionality (93). A study by Cruz and colleagues evaluated PARPi response in 20 breast cancer PDX models harboring g*BRCA1/2* mutations that had demonstrated variable response to PARPi therapy. High RAD51 foci correlated with poor PARPi response, regardless of the causal mechanism restoring HRR function (84). Taken together, these studies support RAD51 foci detection as a robust, functional biomarker capable of capturing real-time HRD status. However, several barriers have prevented clinical validation and implementation, including lack of standardized cutoffs, solid tumor tissue requirements, assay failure rates, and reliance on manual immunofluorescence scoring by trained pathologists (94–97).

### Epigenetic assays: BRCA methylation.

Epigenetic changes may also contribute to HRD tumors, including hypermethylation of *BRCA1* and *RAD51* promoter regions that lead to reduced gene expression. Hypermethylation within the *BRCA1* promoter is estimated to occur in 9%–14% of breast cancers, whereas *RAD51C* promoter hypermethylation remains relatively uncommon (98–101). Notably, *BRCA1* hypermethylation in breast and ovarian cancer is largely mutually exclusive with germline or somatic *BRCA1/2* mutations, suggesting strong selective pressure to inactivate BRCA1 function through either genetic mutation or epigenetic silencing in these tumors (102, 103).

Clinical data supporting the use of *BRCA1* methylation as a biomarker to guide PARPi therapy remain sparse. In a small study of patients with TNBC treated with PARPi-based therapy, those with *BRCA1* promoter methylation or g*BRCA1/2* mutations experienced a significantly improved 6-month PFS compared with patients with low *BRCA1* methylation levels (*P* = 0.009). Notably, 1 patient with high *BRCA1* promoter methylation (>80%) achieved a complete response (104). In contrast, the phase II COMETA-Breast trial, which enrolled 11 patients with advanced TNBC harboring confirmed s*BRCA1/2* promoter methylation in the absence of g*BRCA1/2* mutations, did not meet its predefined efficacy endpoints. Treatment with olaparib resulted in a low ORR of 9% and a mPFS of 2 months (105).

## Mechanisms of resistance to PARP inhibition

While PARPis provide meaningful clinical benefit for many patients, durability of response is frequently limited by the emergence of resistance. Resistance to PARPis can arise through multiple biologically distinct mechanisms. The best-characterized mechanism is restoration of functional HRR, mostly through secondary *BRCA1/2* reversion mutations that restore the open reading frame and protein function (73). Other mechanisms include the emergence of hypomorphic *BRCA1/2* alleles (106) that retain partial DNA repair activity, as well as epigenetic alterations, such as loss of *BRCA1* promoter methylation. Resistance may also result from loss of DNA end resection regulation through alterations in *53BP1*, *REV7*, or components of the Shieldin complex, which permit DNA end resection and enable partial HRR restoration independently of *BRCA1*, or through alterations in *PARP1*, including reduced expression or mutations that diminish PARP trapping, thus attenuating the cytotoxicity of PARP inhibition. Finally, replication fork stabilization and suppression of DNA single-strand gaps, independent of canonical HRR restoration, can protect tumor cells from PARPi-induced DNA damage, and enhanced drug efflux may contribute to therapeutic failure (1, 107, 108).

A comprehensive understanding of these resistance mechanisms is critical for the development of effective and dynamic biomarkers of resistance, as well as the development of next-generation PARPis and rational combination strategies aimed at overcoming resistance and enhancing sensitivity to PARPi agents.

## Novel PARPi combinations

### PARPis with antibody-drug conjugates.

Preclinical studies support the potential utility of combining PARPis with antibody-drug conjugates (ADCs) in breast cancer, particularly those with topoisomerase 1 (TOP1) inhibitor payloads. PARP1 is critical for repairing DNA damage induced by TOP1 inhibition, making it a rational synergistic target (109, 110).

In a phase Ib dose-escalation trial, 30 patients with metastatic TNBC were treated with the trophoblast cell-surface antigen-2–directed (TROP2-directed) ADC sacituzumab govitecan (SG) in combination with talazoparib, either concurrently (*n* = 7) or sequentially (SG followed by talazoparib, *n* = 27). The concurrent dosing strategy resulted in dose-limiting toxicity (particularly myelosuppression) and a mPFS of 2.3 months. In contrast, sequential administration of SG followed by talazoparib was well tolerated, with no dose-limiting toxicities and a mPFS of 7.6 months (111). In the phase II dose-expansion cohort, 26 patients with metastatic TNBC were treated with SG followed by talazoparib, with mandatory G-CSF prophylaxis. The mPFS was 6.2 months, and the median OS was 18.0 months. The confirmed ORR was 30.1%, and the 6-month clinical benefit rate was 53.8%. Serious treatment-related adverse events were observed in 26.9% of participants (112).

First-generation PARPis are relatively nonselective, targeting PARP1, PARP2, and other members of the PARP family, which likely contributes to their broader toxicity profile. PARP2 inhibition has been implicated in the hematologic toxicity observed with pan-PARPis due to its role in erythropoiesis (113), whereas PARP1 appears to mediate the majority of the synthetic lethality underlying the antitumor activity of PARP inhibition (23, 114). Accordingly, next-generation PARPis with greater PARP1 selectivity and a potentially improved hematologic safety profile may represent more suitable partners for combination strategies with ADCs. Nonetheless, even with these novel agents, a sequential rather than concurrent administration strategy is likely preferable to mitigate overlapping toxicities, particularly dose-limiting myelosuppression. Notably, a fundamental limitation of preclinical models evaluating PARPi combinations stems from the use of immunodeficient mice in xenograft studies, which do not exhibit PARPi dose-limiting myelosuppression, likely obscuring the true hematologic effect of these combinations (115).

Preclinical studies have demonstrated antitumor activity with the combination of AZD8205, a B7-H4–directed ADC carrying a TOP1 inhibitor payload, and the PARP1-selective inhibitor saruparib (AZD5305). In a study of 26 PDX models, the combination achieved an ORR of 69%, with responses correlating with HRD status (116). A phase I clinical trial evaluating this combination is ongoing (NCT05123482).

Saruparib is also being evaluated in the phase I/IIa PETRA study, either as monotherapy (117) or in combination with chemotherapy, endocrine therapy, or ADCs, including trastuzumab deruxtecan (NCT04644068), a HER2-directed ADC with a TOP1 inhibitor payload. Additional preclinical data support the combination of saruparib with datopotamab deruxtecan, a TROP2-directed ADC with a TOP1 inhibitor payload. In TNBC (*n* = 5) and non–small cell lung cancer (*n* = 6) cell lines, the combination exhibited significantly enhanced cytotoxic activity compared with either agent alone, achieving up to 95% tumor growth inhibition (118).

SG is also being explored with GS-0201, another PARP1-selective inhibitor, in a first-in-human study in solid tumors (NCT06167317). Additional clinical trials are investigating combinations of both first-generation and PARP1-selective inhibitors with TOP1-based ADCs (NCT04585958, NCT05417594) (Supplemental Table 2).

### PARPis with other targeted therapies.

PARPis in combination with targeted therapy have shown clinical efficacy across a number of therapeutic targets. Multiple preclinical studies suggested that PI3K inhibitors (PI3Kis) increase DNA damage and may act synergistically with PARPis to induce cell death (119–121), although response rates in clinical trials remain modest. A phase I study evaluating olaparib in combination with the PI3Ki BKM120 showed an ORR of 28% and improved outcomes in patients with g*BRCA1/2* mutations (122). Another study of olaparib and alpelisib in a heavily pretreated cohort of patients with TNBC showed an initial ORR of 18%, which improved slightly to 23% at the recommended phase II dose (123). The AKT inhibitor capivasertib was also tested in combination with olaparib, with 44% of patients with advanced breast cancer achieving clinical benefit (RECIST 1.1 CR/PR or stable disease ≥ 4 months) (124).

Antiangiogenic agents have also demonstrated clinical activity in combination with PARPis. The strongest evidence to date comes from the randomized phase III FABULOUS trial, which evaluated the oral PARPi fuzuloparib with or without the VEGF receptor inhibitor apatinib in patients with g*BRCA1/2*-mutated, HER2-negative MBC. The combination achieved an ORR of 67% and a mPFS of 11 months, compared with a mPFS of 6.7 months with fuzuloparib monotherapy (125). In contrast, other VEGF-targeted combination regimens have yielded more modest clinical benefit (126).

Preclinical studies provide a strong rationale for combining PARPis with HSP90 inhibitors and agents targeting cell cycle checkpoint signaling pathways (CHK1, WEE1, and ATR). However, clinical efficacy in breast cancer has not yet been established. HSP90 inhibitor combinations have been evaluated in a small number of patients with breast cancer, with limited activity reported in early studies (127). ATR inhibitor combinations have demonstrated some activity in biomarker-selected populations, such as tumors harboring g*BRCA1/2* and other HRR gene alterations, although this was shown only in a noncomparative phase II setting (128). Ultimately, the randomized phase II VIOLETTE trial showed similar ORR and PFS across olaparib monotherapy, olaparib plus ATR inhibitor, and olaparib plus WEE1 inhibitor in patients with metastatic TNBC, regardless of HRR status (129, 130).

Tumors with HRD have been shown to rely on DNA polymerase-θ to repair DNA double-strand breaks using the error-prone MMEJ pathway (131). Polymerase-θ inhibitors were developed to exploit this synthetic lethality, with promising preclinical activity against HRR-deficient breast and ovarian tumor models and PDXs (132, 133). These preclinical findings have led to the development of several phase I clinical trials to evaluate polymerase-θ inhibitors, alone (NCT05687110) or in combination with PARPis (NCT06560632), in patients with HRD tumors.

The deubiquitinase enzyme USP1 has also been identified as a crucial mediator of DNA repair through translesion synthesis in *BRCA1/2*-mutant tumors. In preclinical studies, selective USP1 inhibitors produced synergistic antitumor activity with PARPis in HRD tumor models and PDXs, including PARPi-resistant models (134, 135). In a phase I clinical trial, the USP1 inhibitor KSQ-4279 demonstrated an acceptable safety profile alone and in combination with olaparib in patients with solid tumors (136).

Sapacitabine is an orally available prodrug of 2′-C-cyano-2′-deoxy-1-β-d-arabino-pentofuranosylcytosine (CNDAC), a deoxycytidine analog that incorporates into DNA, causing single-strand breaks that develop into double-strand breaks when cells progress through a second S phase (137). In preclinical models, CNDAC has shown synergistic activity with PARPis in HRR-deficient tumors (138, 139). In a phase Ib clinical trial, 10 patients with *BRCA1/2*-mutant HER2-negative MBC were treated with a combination of sapacitabine and olaparib. Due to a high rate of hematologic toxicity, the trial was closed before proceeding to phase II, and the maximum tolerated dose and recommended phase II dose were not determined. Nevertheless, the ORR was 50% and the mPFS was 9.7 months, suggesting potential benefit from similar combination strategies designed to avoid hematologic toxicity, such as combination of sapacitabine with a PARP1-selective inhibitor (140).

### PARPis with endocrine therapy.

Preclinical studies suggest that endocrine therapy may induce DNA damage in hormone receptor–positive (HR^+^) breast cancer, potentially sensitizing tumors to PARP inhibition (141). In the OlympiA trial, the combination of adjuvant endocrine therapy and PARP inhibition for patients with early-stage HR^+^/HER2^–^ breast cancer was found to be safe and effective, with no adverse impact from combination treatment (30). Results from the aforementioned PETRA study investigating the novel PARP1-selective inhibitor AZD5305 with the selective estrogen receptor degrader camizestrant showed an ORR of 22.9% and median duration of response of 5.5 months in 38 patients with advanced breast cancer, without new safety signals (142).

HR^+^/HER2^–^ MBC patients with g*BRCA1/2* mutations often experience worse outcomes on first-line endocrine therapy with a CDK4/6 inhibitor than patients with g*BRCA1/2*–wild-type disease (143, 144). Many patients with g*BRCA2* mutations have concurrent *RB1* LOH, leading to CDK4/6 inhibitor resistance (145). In vitro, HR^+^/HER2^–^ breast cancer cell lines with concurrent *BRCA2* and *RB1* loss are resistant to palbociclib but sensitive to olaparib (146). Multiple trials aimed at addressing this important question are ongoing, including the phase I/II HOPE trial investigating olaparib, palbociclib, and fulvestrant combination therapy in patients with MBC and *BRCA1/2* mutations (147), as well as the phase III EvoPAR-BR01 trial evaluating saruparib plus camizestrant in patients with advanced breast cancer and g*BRCA1/2* or g*PALB2* mutations (147).

### PARPis with immunotherapy.

Synergy between PARPis and immunotherapy is postulated to occur through several mechanisms, primarily the activation of the stimulator of interferon genes pathway (148–150) and increased tumor immunogenicity in HRR-deficient tumors characterized by higher tumor mutational burden, increased programmed cell death ligand 1 (PD-L1) expression, and greater tumor-infiltrating lymphocyte (TIL) infiltration (151–153).

Early-phase clinical trials have demonstrated encouraging activity for PARPi-immunotherapy combinations. In the phase II MEDIOLA basket trial, olaparib plus durvalumab achieved a 12-week disease control rate of 80% and a mPFS of 8.2 months among 34 patients with g*BRCA1/2*-mutated MBC, with a favorable safety profile (154). Improved outcomes were observed in patients with elevated PD-L1 expression and higher CD8^+^ TIL levels, suggesting that immune-enriched tumors may be more vulnerable to this approach. Similarly, in the TOPACIO study, niraparib plus pembrolizumab demonstrated an ORR of 21% with higher response rates in patients harboring g*BRCA1/2* or s*BRCA1/2* mutations (ORR 47%) and in PD-L1–positive tumors (ORR 32%) (155); notably, the mPFS reached 8.3 months in the *BRCA1/2*-mutated subgroup.

The open-label TALAVE trial enrolled patients with HER2-negative advanced breast cancer harboring either *BRCA1/2* mutations (*n* = 12) or wild-type *BRCA1/2* (*n* = 12). Patients received a 4-week talazoparib induction followed by concurrent talazoparib and avelumab. Among those with *BRCA1/2*-mutant disease, the ORR was 42%, with a mPFS of 9.3 months. In contrast, no responses were observed in the *BRCA1/2*-wild-type cohort (ORR 0%), with a mPFS of 2.9 months. Correlative analyses demonstrated increased infiltration of macrophages and T cells in *BRCA1/2*-mutant tumors but not in wild-type tumors (156). Importantly, the mPFS observed in these early-phase combination studies appears comparable to, or potentially improved relative to, that historically reported with PARPi monotherapy in landmark MBC trials, such as OlympiAD and EMBRACA, underscoring the need for randomized trials to define the incremental benefit of these combinations (26, 29).

However, randomized data evaluating PARPi-immunotherapy combinations have been less definitive. A phase II trial comparing olaparib monotherapy versus olaparib plus atezolizumab in patients with g*BRCA1/2*-mutated, HER2-negative MBC did not demonstrate a clinically meaningful improvement in PFS with the addition of immunotherapy (157). The DORA study evaluated olaparib with or without durvalumab as maintenance therapy following platinum response in patients with advanced TNBC. The mPFS was improved in both olaparib-containing arms compared with continued platinum therapy. However, the incremental benefit of adding durvalumab to olaparib was not clearly established (158). The KEYLYNK-009 trial suggested a trend toward improved PFS and OS with olaparib plus pembrolizumab compared with chemotherapy plus pembrolizumab in patients with metastatic TNBC harboring tumor *BRCA1/2* mutations, suggesting a potential chemotherapy-sparing maintenance strategy in this population (PFS HR 0.70; OS HR 0.81) (159). Overall, tolerability profiles were favorable, with no new safety signals identified compared with monotherapy studies.

Building on these clinical observations, emerging preclinical data provide additional mechanistic insight into how the tumor immune microenvironment, particularly macrophages, may influence response to PARPi-based combination strategies. In one preclinical study, macrophages were identified as the most abundant immune cell type in tumor specimens from patients with *BRCA1*-mutant TNBC and were significantly more abundant in *BRCA1*-mutant compared with *BRCA1*–wild-type tumors. In a mouse model, olaparib significantly increased macrophage infiltration in *BRCA1*-mutant TNBC, including immunosuppressive CSF1R^+^ macrophages, which limited T cell proliferation and antitumor function. Notably, the combination of olaparib with an anti-CSF1R antibody resulted in enhanced antitumor activity compared with either agent alone, in a CD8^+^ T cell–dependent manner (160). These findings provided the rationale for the phase Ib AXALAP trial, which is evaluating olaparib in combination with the anti-CSF1R antibody axatilimab in patients with HR^+^/HER2^–^ MBC harboring a germline or somatic mutation in *BRCA1/2* or *PALB2*. The trial is ongoing, with the primary objective of evaluating safety and tolerability, as well as determining the maximum tolerated dose and recommended phase II dose of axatilimab with olaparib (161).

## Conclusions and future directions

The clinical benefit of PARPis in breast cancer is well established for breast cancer patients with g*BRCA1/2* mutations in both the adjuvant and metastatic settings. The next critical frontier lies in rationally expanding their use. Growing evidence supports meaningful activity of PARPis in tumors harboring other HRR gene alterations, particularly g*PALB2* and s*BRCA1/2* variants, further underscoring HRD as a broad and biologically relevant therapeutic vulnerability. Prospective trials that include these molecularly defined populations, along with the systematic integration of robust companion diagnostics, such as HRD genomic assays, mutational signatures, and functional biomarkers, will be critical to advance toward a more precise and inclusive definition of HRD.

Currently, acquired resistance to PARPis remains a major unmet need among patients with HRR-deficient breast cancer. Overcoming this resistance will require a variety of targeted combination strategies. Thus far, strategies incorporating PARPis with immunotherapy, targeted agents, or ADCs have demonstrated feasibility and encouraging antitumor activity in selected settings. The development of PARP1-selective inhibitors, together with increasingly biomarker-driven patient selection, offers the potential to enhance the therapeutic index by reducing treatment-related toxicity. If validated in ongoing studies, these advances could broaden the clinical benefit of PARPis. Ultimately, a biologically informed and precision-guided strategy will be required to fully realize the therapeutic potential of PARP inhibition across the spectrum of breast cancer.

## Author contributions

CSW prepared the figures and wrote the manuscript. FL designed the Review and reviewed and edited the manuscript. PAK, GIS, SMT, JEG, and AR reviewed and edited the manuscript. All authors contributed to refinement of the final content and approved the submitted version.

## Conflict of interest

FL has received consulting/advisory board fees from AstraZeneca, Pfizer, and institutional research fees from AstraZeneca, Incyte, Merck, Zentalis, Gilead, and Ideaya. SMT reports consulting or advisory roles for Novartis, Pfizer/SeaGen, Merck, Eli Lilly, AstraZeneca, Genentech/Roche, Eisai, SystImmune/Bristol Myers Squibb, Daiichi Sankyo, Gilead, Blueprint Medicines, Reveal Genomics, Artios Pharma, Stemline/Menarini, Bayer, Jazz Pharmaceuticals, Cullinan Therapeutics, Circle Pharma, Arvinas, BioNTech, Launch Therapeutics, Zuellig Pharma, Ambrx Biopharma/Johnson & Johnson, Bicycle Therapeutics, BeiGene Therapeutics, Mersana, Summit Therapeutics, Avenzo Therapeutics, Aktis Oncology, Celcuity, Boehringer Ingelheim, Samsung Bioepis, Olema Pharmaceuticals, Tempus, Boundless Bio, Denali Therapeutics, Relay Therapeutics, Corcept, Ottima Pharma, and Ellipses Pharma; a scientific advisory board role at VALANX Biotech; institutional research funding from Genentech/Roche, Merck, Exelixis, Pfizer, Eli Lilly, Novartis, Bristol Myers Squibb (BMS), AstraZeneca, NanoString Technologies, Gilead, SeaGen, OncoPep, Daiichi Sankyo, Stemline/Menarini, Jazz Pharmaceuticals, and Olema Pharmaceuticals; and travel support from Eli Lilly, Gilead, Jazz Pharmaceuticals, Pfizer, Roche, and AstraZeneca. PAK reports funding to Dana-Farber Cancer Institute from AstraZeneca, Pfizer, Eli Lilly, Bayer, GlaxoSmithKline (GSK), Tesaro, and Merck KGaA on trials for which he is the principal investigator; he also reports consulting and/or advisory board participation from Immunogen, GSK, Novartis, Alkermes, AstraZeneca, Merck, Pfizer, EMD-Serono, BMS, Artios, and Schrodinger. GIS has research funding from Merck KGaA/EMD-Serono, Artios, Eli Lilly, and Pfizer and has served on advisory boards for Merck KGaA/EMD-Serono, Circle Pharmaceuticals, Concarlo Therapeutics, Schrodinger, FoRx Therapeutics, and MycRx. GIS holds patents titled “Dosage regimen for sapacitabine and seliciclib” and “Compositions and Methods for Predicting Response and Resistance to CDK4/6 inhibition.”

## Supplementary Material

Supplemental data

## Figures and Tables

**Figure 1:**
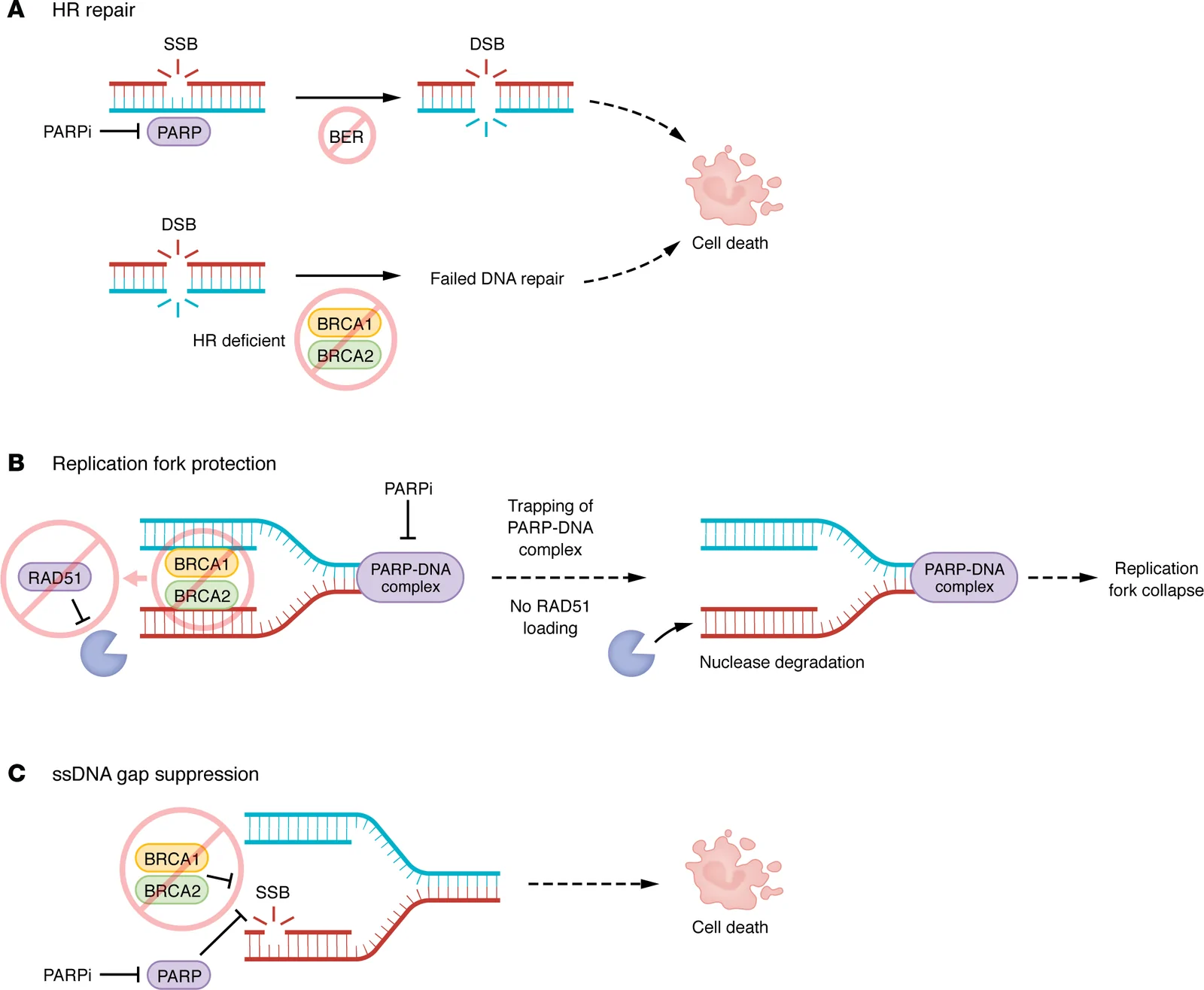
BRCA function in maintenance of genomic stability — lethality with PARP inhibition. Schematic illustrating the key functions of BRCA1/2 in maintaining genomic stability and the mechanisms underlying synthetic lethality with PARP inhibition. BRCA1/2 play critical roles in maintaining genomic integrity through three major mechanisms. (**A**) Homologous recombination–mediated (HR-mediated) repair of DNA double-strand breaks. PARP is recruited to sites of single-strand DNA breaks and facilitates their repair. In HR-deficient cells lacking functional BRCA1/2, double-strand break repair is compromised, resulting in genomic instability and ultimately cell death. (**B**) Protection of stalled replication forks from nucleolytic degradation. BRCA1 and BRCA2 also play essential roles in protecting stalled replication forks from degradation. During replication stress induced by PARPi treatment, PARP-DNA complexes become trapped on DNA, causing replication fork stalling. BRCA1 and BRCA2 facilitate loading of RAD51 onto stalled replication forks, preventing nucleases, such as MRE11, from degrading newly synthesized DNA. In the absence of BRCA1 or BRCA2, RAD51 loading fails to occur, leaving replication forks vulnerable to nucleolytic degradation and resulting in replication fork collapse and cytotoxicity. (**C**) Suppression of single-stranded DNA gap formation during DNA replication. Both PARP and BRCA1/2 suppress single-stranded DNA gap formation during DNA replication. PARP inhibition in BRCA1/2-mutant cells results in loss of both gap suppression mechanisms, leading to the accumulation of single-stranded DNA gaps and ultimately cell death. BER, base excision repair; ssDNA, single-stranded DNA; SSB, single-strand break.

**Figure 2:**
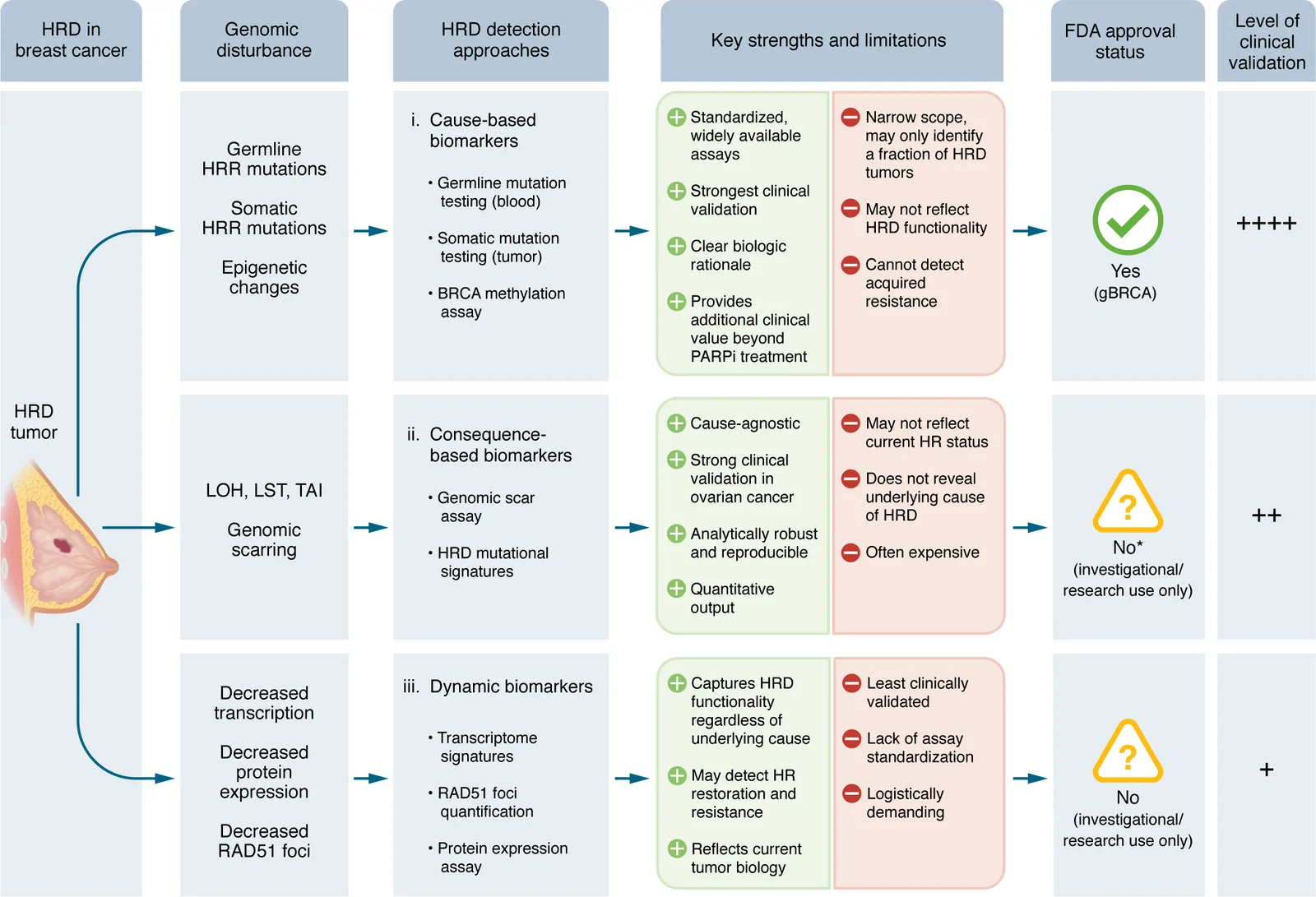
Approaches to HRD detection in breast cancer. A schematic overview of the genomic and functional strategies used to identify HRD in breast tumors. HRD can arise from a variety of mechanisms, including germline and somatic mutations in HR genes such as BRCA1/2 and PALB2, as well as transcriptomic and epigenetic changes. Methods of HRD detection can be broadly categorized as (i) cause-based biomarkers, such as germline or somatic mutation testing, which are supported by strong biological rationale and extensive clinical validation but identify only a subset of HRD tumors; (ii) consequence-based biomarkers that capture genomic scarring, LOH, large-scale transitions, and telomeric allelic imbalance, which offer strong clinical validation in ovarian cancer but are often expensive and may not reflect current HR status; and (iii) dynamic biomarkers that assess real-time functional HR status, such as RAD51 foci detection and transcriptome signatures, which are promising but lack clinical validation and assay standardization. LST, large-scale state transition; TAI, telomeric allelic imbalance. *Myriad Genetics MyChoice CDx is FDA approved as a companion diagnostic for selection of patients with HRD-positive, advanced ovarian cancer who may benefit from first-line maintenance treatment with a PARPi. Although genomic scar assays are widely used in breast cancer research, no HRD assay is currently FDA approved for patient selection in breast cancer.

## Version 1 10/01/2026

Electronic publication
