# Supplementary material for: Expanding the therapeutic scope of PARP inhibitors in breast cancer: current indications and future directions
Source: J Clin Invest. 2026 Oct 1;136(19):e207632. doi: 10.1172/JCI207632 (PMC13626854; doi:10.1172/JCI207632)
Supplement: Supplementary file 1 [file jci-136-207632-s105.pdf]

Online supplemental material regarding the Review manuscript

Expanding the therapeutic scope of PARP inhibitors in breast cancer: current indications and future directions

Charlotte S. Walmsley[1,2], Adela Rodriguez[1,2,3], Panagiotis A. Konstantinopoulos[1,2], Geoffrey I. Shapiro[1,2], Sara M. Tolaney[1,2], Judy E. Garber[1,2,3], Filipa Lynce[1,2]

[1]Department of Medical Oncology, Dana-Farber Cancer Institute, Boston, Massachusetts

[2]Harvard Medical School, Boston, Massachusetts

[3]Division of Cancer Genetics and Prevention, Dana-Farber Cancer Institute, Boston, Massachusetts

Address correspondence to:

Filipa Lynce, 450 Brookline Avenue, Boston, Massachusetts 02215, USA. Phone: 617-632-3000 Email: filipa_lynce@dfci.harvard.edu

Supplemental Table 1. Clinical evidence for use of HRD biomarker assays in breast cancer.

Biomarker	Brief assay description	Detection Method	Sample Type	Regulatory Status	Clinical Investigation	Key results	Advantages	Limitations	Level of Clinical Validation
sBRCA1/2	Somatic tumor testing for *BRCA1* or *BRCA2* pathogenic variants, typically performed through next-generation sequencing	Tumor NGS panel testing	FFPE tumor tissue, blood/cfDNA under investigation	FDA-approved in ovarian and prostate cancer; investigational in breast cancer. Approved for g/sBRCA mutated HER2-negative breast cancer in Japan	UMIN000018721 (1)	ORR higher with s*BRCA* vs non-s*BRCA* (60% vs 33.3%) in patients with advanced TNBC treated with olaparib + eribulin, but did not meet threshold for clinical significance (P=0.264).	Strong biologic rationale, clinically demonstrated activity, FDA approved in other disease types	Tumor heterogeneity and clonal evolution may affect detection and response, requires tumor tissue	Moderate
					NCT02401347 (Olaparib Expanded [TBCRC048]) (2, 3)	Olaparib showed activity in s*BRCA* mBC: ORR 50%, mPFS 6.3 mo; expansion cohort ORR 37%, mPFS 5.6 mo.			
					NCT03990896 (4)	Results not yet available.			
gPALB2	Germline testing for *PALB2* pathogenic variants, often through multigene panel testing	Germline or tumor NGS panel tesing	Blood and/or tumor tissue	Investigational. Olaparib listed by NCCN Breast Cancer Guidelines as "other recommended" (category 2A) in the metastatic setting	NCT02401347 (Olaparib Expanded [TBCRC048]) (2)	Olaparib showed activity in patients with g*PALB2* mBC: ORR 82%, mPFS 13.3 mo; expansion cohort ORR 75%, mPFS 9.6 mo.	Strong biologic rationale, clinically demostrated activity, responses across breast cancer subtypes, NCCN recognition, readily detectable	Low prevalence, no phase III randomized data	Moderate
					NCT02401347 (Talazoparib Beyond BRCA) (5)	Talozaparib showed activity in patients with *gPALB2* mBC: ORR 50%, median PFS 6.9 mo			
Genomic Scar Assay (FoundationFocus CDxBRCA LOH test, myChoice CDx test by Myriad Genetics)	Quantification of large scale chromosomal abnormalities (ex: LOH, TAI, and LST) to create a composite HRD or GIS score	SNP-array or NGS-derived genomic instability scoring	FFPE tumor tissue	FDA-approved as companion diagnostic in ovarian cancer; investigational in breast cancer	NCT00148694, NCT00813956 (PrECOG 0105) - Pooled analysis (6)	HRD score predicted pathologic complete response to platinum chemotherapy in patients wtih TNBC in the neoadjuvant setting, including pts without *BRCA1/2* mutations	Captures broader HRD phenotype beyond *BRCA* and *PALB2* mutations, FDA approved companion diagnostic in ovarian cancer, compatible with existing NGS profiling	May not reflect current HR status, cutoff variability, requires adequate tumor cellularity	Moderate
Mutational signature	Computational methods to quantify abnormal patterns of somatic mutations	Whole-genome sequencing or targeted gene sequencing with computational	Fresh frozen or FFPE tumor tissue	Investigational	EudraCT 2014-003319-12 (RIO) (7)	Patients with HRD-positive TNBC showed decreased ctDNA levels after rucaparib, but no change in Ki67.	Captures broader HRD phenotype beyond *BRCA* and *PALB2*	High cost, bioinformatic complexity, and limited clinical accessibility	Low-to-moderate

	distinct to HRD tumors	signature analysis			Batalini *et al.*, 2023 retrospective real-world database analysis (8)	Patients with HRDsig+ mBC had longer mPFS compared to HRDsig- (6.3 vs 2.8 mo); HR 0.62 (95% CI 0.42–0.92). OS was numerically longer but did not reach statistical significance (17.8 vs 13.0mo, HR 0.72; 95% CI 0.46–1.14)	mutations, can be derived from clinical panel sequencing in some cases (SigMA)		
					NCT01042379 (I-SPY2) (9)	BRCA1ness signature predicted higher pCR in veliparib–carboplatin arm (p=0.03, OR 3.2), not in carboplatin alone (p=0.45) for patients with TNBC in the neoadjuvant setting.			
RAD51 foci detection	Quantification of RAD51 protein accumulation at sites of double stranded DNA breaks	Immunofluorescence microscopy-based RAD51 foci quantification	Fresh or FFPE tumor tissue	Investigational	NCT02789332 (GeparOLA) (post-hoc biomarker analysis) (10)	Low RAD51 score associated with higher pCR to PARPi-based or platinum-based therapies (66.7% vs 22.2% pCR, OR 12.03, 95% CI 2.60–55.73; P=0.002).	Direct functional measurement of HR status, high predictive accuracy in preclinical models, compatible with FFPE tissue	Lack of assay standardization (variable cutoffs, scoring criteria and tissue requirements) requires proliferating cells, uncertain reproducibility due to operator-dependence	Early-stage
					NCT05340413 (RADIOLA) (11)	In patients with g/s *BRCA*, *PALB2*, or *RAD51C/D*–mutated mBC treated with olaparib, ORR was higher in RAD51-low vs RAD51-high tumors (68.3% vs 21.4%, p=0.002).			
BRCA methylation	Epigenetic assay to detect promoter hypermethylation and transcriptional silencing of *BRCA1* gene	Variable approaches including: Methylation-specific PCR, bisulfite pyrosequencing, MS-HRM, ddPCR, or fragment analysis	FFPE or fresh frozen tumor tissue, blood/cfDNA under investigation	Investigational	UMIN000009498 (12)	High *BRCA1* methylation (>80%) or g*BRCA* associated with better 6-month PFS vs low *BRCA1* methylation (p=0.009) in patients with TNBC treated with olaparib plus eribulin	Identifies HRD beyond genetic mutations, stronge biologic rationale, highly correlated with genomic HRD in ovarian cancer, multiple detection methods available	Lack of standardized assays and cutoffs, incomplete methylation may not confer true HRD, limited and conflicting clinical data in breast cancer	Early-stage
					NCT03205761 (COMETA-Breast) (13)	In advanced mTNBC with *sBRCA1/2* methylation, olaparib yielded ORR 9% and mPFS 2 mo; study endpoints not met.			

Supplemental Table 2. Ongoing clinical investigations of PARP inhibitor combination therapies.

Clinical Trial Number	Phase	Class	Early or Advanced	Disease	Agent(s)	Primary End Point	Variant Status	Key Published Findings
NCT04039230	1/2	PARPi + ADC	Advanced	TNBC	Sacituzumab govitecan + talazoparib	DLT	Not specified	Ph 2 (n=26): ORR 30.1%, mPFS 6.2 mo, mOS 18.0 mo
NCT04644068	1/2	PARPi + ADC + endocrine therapy	Advanced	Breast Cancer	AZD5305 + Dato-DXd/T-DXd/camizestrant	DLT, safety	s/g*BRCA1/2*, *PALB2* or *RAD51C/D*	Ph 1/2a (n=38): ORR 22.9%, mPFS 4.4 mo; *BRCAm*/*PALB2m* subset: ORR 33.3%, mPFS 6.1 mo.
NCT05123482	1/2	PARPi + ADC	Advanced	Breast Cancer	AZD8205 + AZD5305	DLT, safety	Not specified	No published results.
NCT04585958	1	PARPi + ADC	Advanced	Solid tumors	Olaparib + trastuzumab deruxtecan	MTD, safety	Not specified	No published results.
NCT06167317	1	PARPi + ADC	Advanced	Solid tumors	GS-0201 + sacituzumab govitecan	DLT, safety	Not specified	No published results.
NCT05417594	1/2	PARPi + ADC	Advanced	Solid tumors	AZD9574 + T-DXd/ Dato-DXd	DLT, safety	s/g*BRCA1/2*, *PALB2* or *RAD51C/D*	No published results.
NCT05498155	2	PARPi + Immunotherapy	Early	TNBC	Olaparib ± durvalumab	pCR	s/g*BRCA1/2*	Ph 2: olaparib mono (n=25) pCR 68%; olaparib + durvalumab (n=25) pCR 80%
NCT05761470	2	PARPi + Immunotherapy	Early	ERBB2-negative BC	Fluzoparib + camrelizumab + chemotherapy	pCR	HRR gene mutation	No published results.
NCT04584255	2	PARPi + Immunotherapy	Early	ERBB2-negative BC	Niraparib + dostarlimab	TILs, pCR	g*BRCA1/2*, g*PALB2*	No published results from this trial. Related pilot (NCT03329937): neoadjuvant niraparib monotherapy in g*BRCA* BC showed pCR 40% (6/15).
NCT04837209	2	PARPi + Immunotherapy	Advanced	TNBC	Niraparib + dostarlimab + radiation therapy	ORR	Not specified	No published results.
NCT02734004	1/2	PARPi + Immunotherapy	Advanced	ERBB2-negative BC	Olaparib + durvalumab	DCR, ORR, safety	g*BRCA1/2*	Ph 1/2 (n=30, g*BRCA* MBC): 12-wk DCR 80%, 28-wk DCR 50%, mPFS 8.2 mo.
NCT02849496	2	PARPi + Immunotherapy	Advanced	ERBB2-negative BC	Olaparib + atezolizumab	PFS	*BRCA1/2*	No significant PFS benefit for combination vs. olaparib alone in g*BRCA1/2* advanced BC.
NCT03740893	2	PARPi + Immunotherapy	Early	TNBC	Olaparib + durvalumab	Ki67, ctDNA, TILs, gene expression signature	g*BRCA1/2*, HRD	No published results.
NCT05759546	2	PARPi + CDK4 inhibitor + endocrine therapy	Advanced	HR+/ERBB2-negative BC	Fluzoparib + dalpiciclib+ fulvestrant/AI	PFS	Not specified	No published results.
NCT05582499	1/2	PARPi + CDK inhibitor	Early	Breast cancer	Fluzoparib + dalpiciclib/ fluzoparib + chemotherapy	pCR	Not specified	Ph 2 (n=251): AI-guided precision tx pCR 11.1% vs control 4.0% (P=0.033); targeted subgroup pCR 13.9% vs 4.0% (P=0.026)
NCT05594095	2	PARPi + CDK inhibitor	Advanced	HR+/ERBB2-negative BC	Fluzoparib + dalpiciclib	ORR	*BRCA1/2*, *PALB2*	No published results in BC. RP2D established in sarcoma (FOUNDS).
NCT04503265	1/2	PARPi/MP inhibitor	Advanced	Solid tumors	AMXI-5001	MTD, RP2D, safety	HRD	No published results.
NCT03162627	1	PARPi + MEK inhibitor	Advanced	Solid tumors	Olaparib + selumetinib	MTD	Not specified	No published results.

NCT05898399	1/2	PARPi + Pol-θ inhibitor	Advanced	Solid tumors	ART6043 ± olaparib/ talazoparib	DLT, PFS	s/g*BRCA1/2*	Ph 1 dose escalation (n=61, solid tumors): ART6043 well-tolerated alone and with olaparib. Anti-tumor activity signals in combination
NCT05564377	2	PARPi + PIK3CA inhibitor	Advanced	Solid tumors	Olaparib + alpelisib	Accrual rate	Not specified	Predecessor Ph 1b (NCT01623349, TNBC, n=17): ORR 18%, DCR 59%, mDOR 7.4 mo. Activity in non-*BRCA* carriers.
NCT05358639	1	PARPi + BCL2 inhibitor	Advanced	TNBC	Olaparib + navitoclax	RP2D	s/g*BRCA1/2* or *PALB2*	Trial design abstract only. No efficacy data reported.
NCT05759546	2	PARPi + CDK inhibitor + endocrine therapy	Advanced	HR+/ERBB2-negative BC	Fluzoparib + dalpiciclib + fulvestrant/AI	PFS	Not specified	No published results.
NCT06612814	3	PARPi + CDK inhibitor + endocrine therapy	Advanced	HR+/ERBB2-negative BC	Fluzoparib + dalpiciclib + fulvestrant/AI	PFS	Not specified	No published results.
NCT03368729	1/2	PARPi + HER2/neu receptor antagonist	Advanced	ERBB2-positive BC	Niraparib + trastuzumab	DLT, ORR	Not specified	Ph 1b/2 (n=41, HER2+ MBC): CBR 44%. Manageable safety. B-cell/TLS signature enriched in responders
NCT01116648	1/2	PARPi + VEGFR inhibitor	Advanced	TNBC	Olaparib + cediranib	DLT, MTD, PFS	Not specified	Ph 1 (n=28; 7 BC evaluable): RP2D established. No objective responses in BC; 2 SD >24 wk. Gr≥3 AEs 75%
NCT04090567	2	PARPi + ATR inhibitor + VEGFR inhibitor	Advanced	Breast cancer	Olaparib + cediranib/ ceralasertib	ORR	g*BRCA1/2*	Ph 2 (n=15, post-PARPi g*BRCA* MBC): ORR 13.3%, CBR 26.7%. Manageable toxicity.
NCT03330847	2	PARPi + ATR inhibitor + WEE1 inhibitor	Advanced	TNBC	Olaparib ± ceralasertib/ adavosertib	PFS	Not specified	Ph 2 (mTNBC): No PFS improvement with combinations vs. olaparib alone across *BRCA*m, non-*BRCA* HRRm, and HRR-WT strata. Similar ORR across arms.
NCT06488378	1	PARPi + CSF-1R inhibitor	Advanced	ERBB2-negative BC	Olaparib + axatilimab	MTD, RP2D	s/g*BRCA1/2*, g*PALB2*	No published results.
NCT06380751	3	PARPi + CDK inhibitor + endocrine therapy	Advanced	HR+/ERBB2-negative BC	AZD5305 + CDK4/6 /Camizestrant	PFS	g*BRCA1/2*, gPALB2	No published results.
NCT06201234	2	PARPi + endocrine therapy	Advanced	HR+/ERBB2-negative BC	Olaparib ± elacestrant	PFS	g*BRCA1/2*	No published results.
NCT03742245	1	PARPi + HDAC inhibitor	Advanced	Breast cancer	Olaparib + vorinostat	MTD	Not specified	No published results.

Supplementary Table 2 Footnote: Trials were identified through a systematic search of ClinicalTrials.gov using the terms 'PARP inhibitor' AND 'breast cancer' combined with each combination class (e.g., ADC, immunotherapy, CDK inhibitor). Inclusion criteria were: (1) interventional trials combining a PARP inhibitor with at least one other anticancer agent; (2) enrollment of patients with breast cancer or solid tumors inclusive of breast cancer; (3) active or recruiting as of 2/1/2026.. Phase I monotherapy dose-finding studies without a combination partner were excluded. Published results were

identified through PubMed and conference abstract databases (ASCO, ESMO, SABCS, AACR).

Supplemental references

1. Shimomura A, Yonemori K, Yoshida M, Yoshida T, Yasojima H, Masuda N, et al. Gene Alterations in Triple-Negative Breast Cancer Patients in a Phase I/II Study of Eribulin and Olaparib Combination Therapy. *Transl Oncol.* 2019;12(10):1386-94.

2. Tung NM, Robson ME, Ventz S, Santa-Maria CA, Nanda R, Marcom PK, et al. TBCRC 048: Phase II Study of Olaparib for Metastatic Breast Cancer and Mutations in Homologous Recombination-Related Genes. *Journal of Clinical Oncology.* 2020;38(36):4274-82.

3. Tung NM, Robson ME, Nanda R, Li T, Vinayak S, Shah PD, et al. TBCRC 048 (olaparib expanded) expansion cohorts: Phase 2 study of olaparib monotherapy in patients (pts) with metastatic breast cancer (MBC) with germline (g) mutations in PALB2 or somatic (s) mutations in BRCA1 or BRCA2. *Journal of Clinical Oncology.* 2024;42(16_suppl):1021-.

4. Vidula N, Damodaran S, Bhave MA, Rugo HS, Blouch E, Ruffle-Deignan NR, et al. Phase II study of a PARP inhibitor in somatic <i>BRCA1/2</i> mutant metastatic breast cancer (MBC). *Journal of Clinical Oncology.* 2023;41(16_suppl):TPS1134-TPS.

5. Gruber JJ, Afghahi A, Timms K, DeWees A, Gross W, Aushev VN, et al. A phase II study of talazoparib monotherapy in patients with wild-type BRCA1 and BRCA2 with a mutation in other homologous recombination genes. *Nat Cancer.* 2022;3(10):1181-91.

6. Telli ML, Timms KM, Reid J, Hennessy B, Mills GB, Jensen KC, et al. Homologous Recombination Deficiency (HRD) Score Predicts Response to Platinum-Containing Neoadjuvant Chemotherapy in Patients with Triple-Negative Breast Cancer. *Clin Cancer Res.* 2016;22(15):3764-73.

7. Chopra N, Tovey H, Pearson A, Cutts R, Toms C, Proszek P, et al. Homologous recombination DNA repair deficiency and PARP inhibition activity in primary triple negative breast cancer. *Nat Commun.* 2020;11(1):2662.

8. Batalini F, Madison RW, Sokol ES, Jin DX, Chen KT, Decker B, et al. Homologous Recombination Deficiency Landscape of Breast Cancers and Real-World Effectiveness of Poly ADP-Ribose Polymerase Inhibitors in Patients With Somatic BRCA1/2, Germline PALB2, or Homologous Recombination Deficiency Signature. *JCO Precis Oncol.* 2023;7:e2300091.

9. Severson TM, Wolf DM, Yau C, Peeters J, Wehkam D, Schouten PC, et al. The BRCA1ness signature is associated significantly with response to PARP inhibitor

treatment versus control in the I-SPY 2 randomized neoadjuvant setting. *Breast Cancer Res.* 2017;19(1):99.

10. Villacampa G, Llop-Guevara A, Filmann N, Herencia-Ropero A, Fasching PA, Karn T, et al. RAD51 Testing in Patients with Early HER2-Negative Breast Cancer and Homologous Recombination Deficiency: A Post Hoc Analysis of the GeparOLA Trial. *Clin Cancer Res.* 2025;31(5):808-14.

11. Pimentel I, Pascual T, Lema L, Tolosa P, Adamo B, Martínez MT, et al. Abstract RF5-05: Predicting olaparib sensitivity in patients with metastatic HER2-negative breast cancer with BRCA1/2, PALB2, RAD51C/D mutations according to their homologous recombination status by the RAD51 test: Primary analysis of the RADIOLA phase II trial. *Clinical Cancer Research.* 2026;32(4_Supplement):RF5-05-RF5-.

12. Kawachi A, Yamashita S, Okochi-Takada E, Hirakawa A, Tsuda H, Shimomura A, et al. BRCA1 promoter methylation in breast cancer patients is associated with response to olaparib/eribulin combination therapy. *Breast Cancer Res Treat.* 2020;181(2):323-9.

13. Haba JDL, Anton A, Quiroga V, Guerrero A, Andrés R, Mele M, et al. Olaparib (O) in advanced triple negative breast cancer (aTNBC) patients (pts) with BRCA1/2 promoter methylation: GEICAM/2015-06 study (COMETA-Breast). *Journal of Clinical Oncology.* 2023;41(16_suppl):1093-.
